# Admixture Has Shaped Romani Genetic Diversity in Clinically Relevant Variants

**DOI:** 10.3389/fgene.2021.683880

**Published:** 2021-06-16

**Authors:** Neus Font-Porterias, Aaron Giménez, Annabel Carballo-Mesa, Francesc Calafell, David Comas

**Affiliations:** ^1^Departament de Ciències Experimentals i de la Salut, Institut de Biologia Evolutiva (UPF-CSIC), Universitat Pompeu Fabra, Barcelona, Spain; ^2^Facultat de Sociologia, Universitat Autònoma de Barcelona, Barcelona, Spain; ^3^Facultat de Geografia i Història, Universitat de Barcelona, Barcelona, Spain

**Keywords:** Romani, whole-exome sequences, clinically relevant variants, drug-response variants, local ancestry inference, Eurocentric bias

## Abstract

Genetic patterns of inter-population variation are a result of different demographic and adaptive histories, which gradually shape the frequency distribution of the variants. However, the study of clinically relevant mutations has a Eurocentric bias. The Romani, the largest transnational minority ethnic group in Europe, originated in South Asia and received extensive gene flow from West Eurasia. Most medical genetic studies have only explored founder mutations related to Mendelian disorders in this population. Here we analyze exome sequences and genome-wide array data of 89 healthy Spanish Roma individuals to study complex traits and disease. We apply a different framework and focus on variants with both increased and decreased allele frequencies, taking into account their local ancestry. We report several OMIM traits enriched for genes with deleterious variants showing increased frequencies in Roma or in non-Roma (e.g., obesity is enriched in Roma, with an associated variant linked to South Asian ancestry; while non-insulin dependent diabetes is enriched in non-Roma Europeans). In addition, previously reported pathogenic variants also show differences among populations, where some variants segregating at low frequency in non-Roma are virtually absent in the Roma. Lastly, we describe frequency changes in drug-response variation, where many of the variants increased in Roma are clinically associated with metabolic and cardiovascular-related drugs. These results suggest that clinically relevant variation in Roma cannot only be characterized in terms of founder mutations. Instead, we observe frequency differences compared to non-Roma: some variants are absent, while other have drifted to higher frequencies. As a result of the admixture events, these clinically damaging variants can be traced back to both European and South Asian-related ancestries. This can be attributed to a different prevalence of some genetic disorders or to the fact that genetic susceptibility variants are mostly studied in populations of European descent, and can differ in individuals with different ancestries.

## Introduction

Human genetic diversity is a continuum, which means that there are no fixed, immutable or discrete boundaries between populations. Linguistic, geographic and social factors can lead to different demographic histories and, in turn, to patterns of inter-population variability (Lao et al., 2008; Barbujani et al., 2013; Batai et al., 2020; Heyer and Reynaud-Paligot, 2020). It has been previously reported that this stratification has consequences in the genetics of complex traits and diseases [reviewed in Bentley et al. (2017); Sirugo et al. (2019)]. On the one hand, disease-associated variants present allele frequency differences across populations. For example, 70% of the cystic fibrosis cases in Europeans are due to ΔF508 mutation in *CFTR* gene, while the most common causal variant in South Africans with African ancestry is 3120 + 1G→A, and different mutations have different therapeutic targets (Padoa et al., 1999; Stewart and Pepper, 2017; Sirugo et al., 2019). On the other hand, genomic variation across populations is also observed for treatment response differences, especially in genes related to absorption, distribution, metabolism, and excretion (ADME) of drugs (Dopazo et al., 2016; Škarić-Jurić et al., 2018; Sirugo et al., 2019). For example, the metabolism of the anticoagulant warfarin can differ due to several genetic polymorphisms; however, their frequencies are different in European and African descent groups, which challenges the correct dosage prescription (Bress et al., 2012; Johnson et al., 2017; Sirugo et al., 2019).

Thus, an accurate clinical assessment relies on the study of clinically relevant genetic variants with different allele frequencies across groups. Yet, there is an underrepresentation of human populations in the screening of these variants. Particularly, genetic studies show a strong and systematic Eurocentric bias (Need and Goldstein, 2009; Popejoy and Fullerton, 2016; Bentley et al., 2017; Martin et al., 2019; Sirugo et al., 2019). As a consequence, this bias prevents to fully understand the genetic architecture of human disease and leads to an incomplete genetic assessment of complex traits, and to an inaccurate disease diagnosis and treatment in under-represented groups (Martin et al., 2019; Sirugo et al., 2019).

The Roma population, also known by the misnomer of “Gypsies,” has been under-represented in these genome-wide scans. They constitute the largest transnational minority ethnic group in Europe (Council of Europe, 2012). Linguistic and genetic evidence point to a South Asian origin and subsequent diaspora toward Europe, with extensive non-Roma gene flow and multiple founder effects shaping their demographic history (Hancock, 2002; Matras, 2002; Mendizabal et al., 2012; Moorjani et al., 2013; Morar et al., 2013; Font-Porterias et al., 2019). Most medical genetic studies on this population have been focused on targeting the genetic variants responsible for the increased prevalence of certain genetic diseases [reviewed in Kalaydjieva et al. (2001); Morar et al. (2013)]. In this sense, several founder mutations have been identified: e.g., the p.R299X mutation in the *LTBP2* gene, which is responsible for congenital glaucoma (Azmanov et al., 2011). However, the distribution of disease-associated variants in this population has not been fully characterized. In addition, drug response-related genome-wide variation has only been deeply examined in Croatian Roma, where variants in ADME genes were found to have increased allele frequencies (Škarić-Jurić et al., 2018).

To fill this gap, we examine whole exome sequences (WES) and genome-wide array data of 89 healthy Spanish Roma individuals and characterize the functionally relevant genomic variants (i.e., associated to disease or to drug response) with either increased or decreased allele frequencies in the Roma. Beyond frequency distribution differences, and taking into account that Roma is an admixed population, we describe the ancestral origin of multiple variants by leveraging on the estimated local ancestry of their background haplotypes.

## Materials and Methods

### Data

We used WES (mean depth of 54X), and genome-wide autosomal SNP data (Affymetrix Axiom Genome-Wide Human Origins 1 array) for Spanish Roma individuals (89 and 62 samples, respectively) (Font-Porterias et al., 2021), deposited at EGA (EGAS00001004599). The Spanish Roma WES were merged with previously published non-Roma WES from 1000G (mean depth of 65.7X): Iberian Population in Spain (IBS), Toscani in Italia (TSI), Punjabi from Lahore (PJL), Indian Telugu from the United Kingdom (ITU), and Gujarati Indian from Houston (GIH) (Auton et al., 2015), resulting in a dataset with 512 individuals and 410,225 variants. The genome-wide SNP data was merged with IBS, TSI, PJL, ITU, and GIH from 1000G (1000 Genomes Project Consortium, 2012), with 474,632 genome-wide SNPs in 487 samples. Both datasets were then combined to increase the covered genomic variants, building a merged WES-array dataset with 487 individuals and 878,162 SNPs. Variant annotation was performed using the Variant Effect Predictor tool (VEP) from Ensembl (McLaren et al., 2016) focusing on three deleterious prediction scores: PolyPhen-2 (Adzhubei et al., 2010), GERP (Davydov et al., 2010) and CADD (Rentzsch et al., 2019), as previously explained (Font-Porterias et al., 2021). For each analysis, the corresponding dataset used is specified (WES dataset or merged WES-array dataset). In addition, we have included a glossary of terms that may have ambiguous meanings in genetic studies (Supplementary Note 1).

### Local Ancestry Inference

The phasing of the merged WES-array dataset, with 405,814 variants with minor allele frequency (MAF) > 1%, was performed using SHAPEIT (O’Connell et al., 2014), using the population-averaged genetic map from the HapMap phase II (International HapMap Consortium, 2003) and the 1000G dataset as a reference panel (1000 Genomes Project Consortium, 2012). RFMix v1.5.4 (Maples et al., 2013) was run with one expectation-maximization (EM) iteration to infer the local ancestry of the phased haplotypes, using balanced reference panels representing European (IBS and TSI populations) and South Asian (PJL, GIH, and ITU populations) ancestries. As previously explained (Font-Porterias et al., 2021), the Roma individuals included in the present study show, on average, 68.4% and 31.6% of European and South Asian global ancestry proportions, with a standard deviation of 7%. Ancestry was assigned when RFMix posterior probability was higher than 0.9, resulting in 96.3% of the variants with assigned ancestry. In order to match the local ancestry inference in heterozygous variants and obtain the ancestry background of the allele, we adjusted the RFMix rephasing as previously performed (Browning et al., 2018), since RFMix partially rephases the data when assigning local ancestry. However, when the variant was filtered out (MAF < 1%) in the phasing, only genotype ancestries can be retrieved.

### Genetic Portability

We computed the allele sharing ratio (proportion of variants at different frequency bins) from the Roma segregating in non-Roma populations from WES variants dataset. In addition, we compared the linkage disequilibrium (LD) decay patterns between Roma and non-Roma from the genome-wide array dataset using PopLDdecay (Zhang et al., 2018) with default parameters. We performed a two-sample Kolmogorov–Smirnov test to check whether the decay distributions of Roma and non-Roma groups were statistically different. For both analyses, we used the same number of individuals per population to avoid sample size biases (70 individuals per population for the allele sharing ratio and 62 individuals per population for the LD decay).

### Gene Enrichment Analyses

Using the WES dataset, we performed two different gene enrichment analyses using WEB-based GEne SeT AnaLysis Toolkit (Liao et al., 2019) to identify categories (or classes) of genes that are over-represented in a particular set of genes, using a background gene set. First, we interrogated those genes with more deleterious mutations (i.e., deleterious N_*alleles*_ or N_*hom*_ per individual per gene). Although we expect that the most constrained genes (i.e., lower values of deleterious N_*alleles*_ or N_*hom*_ per individual per gene) will be shared across populations, we test whether there is a particular pathway enriched in the most mutated genes in the Roma samples and the non-Roma groups, independently. To do so, we normalized N_*alleles*_ or N_*hom*_ by the number of variants in each gene and we then examined the correlation between each pair of Roma to non-Roma populations per each gene. The over-representation analysis was performed with default parameters (Liao et al., 2019), Gene Ontology (GO) was selected as the functional database, and all the genes included in our variants set was used as background gene set. This analysis does not take into account the frequency of the variants, since the calculation is performed per individual. We also performed a gene enrichment analysis to test whether the genes with deleterious variants showing allele frequency increases in Roma to non-Roma (or non-Roma to Roma) belong to specific genetic disease clusters. We included in the analysis those genes with variants with a fold increase in minor allele frequency (MAF) > 5 or fold increase in minor allele count (MAC) > 5 for monomorphic variants. By using this restrictive threshold, we decrease the number of false positive results in the enrichment analyses, although we may lose some pathogenic variants found at low frequencies (and for that reason, we then focus on ClinVar pathogenic variants with less conservative thresholds). The same number of individuals were considered for this analysis (70 individuals per population) to avoid biases in MAC and MAF calculations. The over-representation analysis was performed with default parameters (Liao et al., 2019), OMIM was selected as the functional disease database and all the genes included in our variants set was used as background gene set. Once the enriched pathways were identified, we computed a chi-squared test to check whether the associated genetic variants described in OMIM present in our dataset have statistically different genotype frequencies between Roma and non-Roma groups.

### Screening of Known Disease-Associated Variants

We first identified previously reported Mendelian mutations in the Roma, annotated in Bianco et al. (2020) and checked the ancestry of their haplotypes to trace their putative origin. However, this approach does not allow us to examine if there is a different frequency spectrum of disease-associated variants comparing Roma and non-Roma. To that end, we then annotated the set of WES variants using ClinVar database (Landrum et al., 2014) and compared the frequency of clinically validated variants among populations. We kept only variants with a clinical significance of “pathogenic,” which is the highest level of supported evidence. We selected those variants with a fold increase in risk allele frequency (RAF) > 1.5 between populations or 1.5% RAF for monomorphic variants. A chi-squared test was performed to test whether the genotype frequencies were significantly different across populations. The same number of individuals were considered for this analysis (70 individuals per population) to avoid biases in RAF calculations.

### Screening Beyond Disease-Associated Variants

To examine pharmacogenetic variation in the WES dataset, we studied mutations that disrupt drug binding domains without being deleterious to the protein. These variants are based on function prediction: they might cause drug binding inhibition; however, not all variants have a reported association with drug response (Hopkins and Groom, 2002; Dopazo et al., 2016). We also examined variants found in 31 core ADME genes^1^ (Škarić-Jurić et al., 2018). We selected those variants with a fold increase in MAF > 1.5 between populations or 1.5% MAF for monomorphic variants. A chi-squared test was performed to test whether the genotype frequency was significantly different across populations. The same number of individuals were considered for this analysis (70 individuals per population) to avoid biases in MAF calculations. The selected variants in both analyses were searched in PharmGKB (Whirl-Carrillo et al., 2012) and the corresponding target drugs in DrugBank (Wishart et al., 2018) and PubChem (Kim et al., 2019).

## Results

### Initial Assessment of Functional Variants as a First Evidence of Inter-Population Variability

**FIGURE 1:**
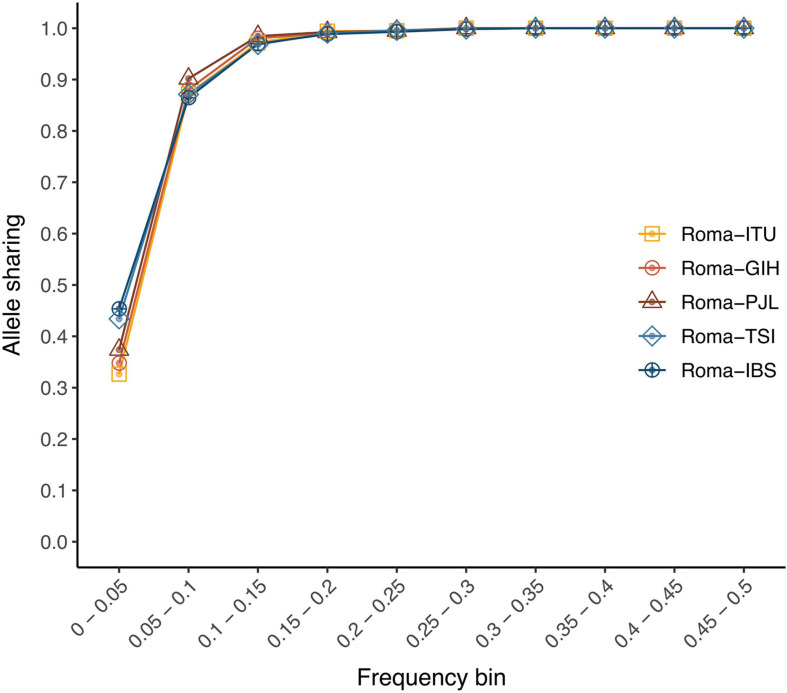
Allele-sharing ratios among Roma and non-Roma groups. Proportion of Roma variants from each minor allele frequency bin (from >0% to 50%) which are also segregating at each non-Roma population.

In order to assess the genetic portability from non-Roma to Roma, we examined the allele sharing and linkage disequilibrium patterns between populations. We have previously shown that Roma exhibit a considerable amount of private variants; however, their proportion is lower than in other populations: 15,287 population-specific variants in Roma; 25,060 in IBS; 24,158 in TSI; 21,160 in PJL; 22,040 in GIH; and 24,070 in ITU (Font-Porterias et al., 2021). In addition, allele sharing is high for common variants (MAF > 5%) (over 86% of Roma variants are present in non-Roma) (Figure 1). However, for rare variants (MAF < 5%), the allele sharing is around 30–40% (Figure 1). Regarding linkage disequilibrium, decay patterns are not statistically different between Roma and non-Roma (Supplementary Figure 1, *p*-value > 0.95). The total number of deleterious alleles per individual is similar among Roma and non-Roma groups (Font-Porterias et al., 2021), and here we further show that the genomic distribution of accumulation of deleterious mutations (i.e., number of deleterious alleles per gene per individual) has comparable patterns between populations (Supplementary Note 2, Supplementary Figures 2, 3, and Supplementary Tables 1, 2). Thus, the overall allele sharing and linkage disequilibrium patterns are comparable among populations. However, rare and private variants can present some challenges in the genetic characterization of Roma population, especially for those variants with a frequency lower than 5%.

As shown in a previous study, Roma and non-Roma groups exhibit differences in the site frequency spectrum due to different demographic histories (Font-Porterias et al., 2021). An overrepresentation analysis including those genes with different allele frequency variants points to a differential OMIM trait enrichment in non-Roma and Roma (Supplementary Note 3 and Supplementary Tables 1, 3). Regarding rare conditions, the genes related to non-Herlitz type junctional epidermolysis bullosa are enriched when comparing deleterious variants with higher frequency in Roma than non-Roma. The prevalence of this disease in Roma is higher than non-Roma in Spain (Martinez-Frias and Bermejo, 1992), although none of the pathogenic variants described in OMIM are present in our dataset and only benign ones show increased frequencies (Supplementary Table 4). In addition, the tetralogy of Fallot is enriched in non-Roma (Table 1), with two pathogenic variants in *GATA4* gene (rs56208331 and rs115099192) with increased frequencies in South Asian groups (Supplementary Table 4). This is consistent with the higher prevalence of this condition in Asia (Takkenberg and Roos-hesselink, 2011).

**TABLE 1:** Summary results of the overrepresentation analysis.

**Type**	**Name**	**Comparison**	**Associated variants**
Rare	Non-Herlitz Epidermolysis Bullosa junctional (OMIM 226650)	Roma > non-Roma	–
Rare	Tetralogy of Fallot (OMIM 187500)	Non-Roma > Roma	rs56208331, rs115099192 (pathogenic) (Tomita-Mitchell et al., 2007; Zhang et al., 2008)
Cardiovascular and metabolic	Obesity (OMIM 601665)	Roma > non-Roma	rs2282440 (associated) (Ha et al., 2006)
Cardiovascular and metabolic	Ischemic stroke (OMIM 601367)	Roma > non-Roma	rs6025 (risk factor) (Majerus, 1994; Casas et al., 2004)
Cardiovascular and metabolic	Insulin dependent diabetes (OMIM 125853)	Roma > non-Roma	–
Cardiovascular and metabolic	Non-insulin dependent diabetes (OMIM 222100)	Non-Roma > Roma	rs1800467 (likely benign) (Phani et al., 2014)
Other	Protection alcohol dependence (OMIM 103780)	Roma > non-Roma	rs1229984 (associated) (MacGregor et al., 2009)
Other	Breast cancer (OMIM 114480)	Non-Roma > Roma	–

*Conditions (traits or diseases) are grouped in three categories: rare, cardiovascular/metabolic and other. Name and Phenotype MIM number from OMIM database are shown. Comparison shows the first population as the one with increased allele frequency variants. Associated variants with increased allele frequencies are shown with its clinical annotation and corresponding reference study. More details can be found in Supplementary Tables 3–6.*

Multiple cardiovascular and metabolic disorders are also present in the gene overrepresentation analysis. Obesity is enriched comparing Roma with non-Roma, especially with Europeans. One variant in the *SDC3* gene (rs2282440), which is associated with obesity in Asians (Table 1) has a significantly increased frequency in Roma (19%) and it is virtually absent in other European groups (Supplementary Tables 4, 5). All risk alleles of this variant in the Roma have South Asian ancestry (Supplementary Table 6), suggesting that this allele in the Roma has a South Asian origin. In addition, genes related to ischemic stroke are overrepresented in Roma (Table 1). One variant in the *F5* gene, annotated as a risk factor in Europeans (rs6025), has significantly higher frequencies in Roma (8.6%) compared to all non-Roma (<1.5%) (Supplementary Tables 4, 5). This variant has a European ancestry background for 7 out of 8 risk alleles found (Supplementary Table 6), suggesting a European origin of this variant in the Roma. Lastly, both types of diabetes are enriched in our analysis: non-insulin dependent diabetes is enriched in non-Roma, while insulin dependent diabetes is overrepresented in Roma compared to non-Roma (Table 1). These results are consistent with previous literature (Mendizabal et al., 2013; Werissa et al., 2019), although our dataset does not include none of the pathogenic or risk-factor variants (more details in Supplementary Note 3).

Other conditions such as protection to alcohol dependence are enriched in Roma compared to non-Roma (Table 1). One variant in the *ADH1B* gene reported to be protective for alcohol dependence (rs1229984) shows a significantly higher allele frequency in Roma without a clear ancestry origin (Supplementary Tables 5, 6). The enrichment for breast cancer in non-Roma (Table 1) is due to variants with increased allele frequencies in *ATM*, *BRCA2*, and *NQO2* genes. Roma show higher prevalence of triple negative (TN) breast tumors (Reckova et al., 2017), and mutations in the enriched genes are mostly linked to non-TN types (Peshkin et al., 2010; Lin et al., 2016; Decker et al., 2017; Slavin et al., 2017; Sirisena et al., 2018).

These results show multiple OMIM traits enriched for genes with a 5-fold increased allele frequency deleterious variants both in Roma and in non-Roma. Although this is an exploratory approach, it is consistent with previous literature and reports the first evidence that Roma do not show a systematically increased genetic susceptibility to disease.

### A Genome-Wide Screening Does Not Support an Increased Susceptibility for Genetic Disorders

The presence of mutations responsible for particular Mendelian disorders in the Roma has been reported in several studies (see (Kalaydjieva et al., 2001; Álvarez et al., 2005; Bouwer et al., 2007; Gamella et al., 2013; Sevilla et al., 2013; Rocha et al., 2014; Cabrera-Serrano et al., 2018) among others). From these previously reported mutations, we found seven variants in our dataset (Table 2). Only in two out of seven variants, the risk allele is present in non-Roma populations: rs1801968 (chr9:132580901) and rs1126809 (chr11:89017961) (Table 2). The risk allele of the latter has a higher frequency in Europeans than South Asians (Table 2) and, in the Roma, it has a European assigned ancestry in 17 out of 19 alleles (Supplementary Table 7), suggesting that this variant (responsible for Oculocutaneous albinism) in the Roma has a European origin, as previously identified (Bianco et al., 2020). In addition, two variants responsible for Charcot–Marie–Tooth disease (rs119483085; chr8:134270617 and rs80338934; chr5:148389835) (Table 2) both have one risk allele with European ancestry (Supplementary Table 7). On the contrary, rs77931234 (chr1:76226846; Acetyl-coA dehydrogenase deficiency) and rs104894396 (chr13:20763650; Deafness) appear to have a South Asian origin (Supplementary Table 7). In fact, rs104894396 risk allele is also present in PJL population (Table 2), which is consistent with a South Asian origin. This variant, only present in Roma and Punjabi individuals in our dataset, is a non-synonymous mutation (W42X) responsible for autosomal recessive non-syndromic hearing loss. It has been previously reported in Spanish and Slovak Roma individuals and an Indian origin has been suggested (Minárik et al., 2003; Álvarez et al., 2005), which is congruent with the South Asian ancestry assignation of the risk allele.

**TABLE 2:** List of previously reported Mendelian mutations in Roma present in this study.

**Variant – allele**	**Gene**	**Roma**	**IBS**	**TSI**	**PJL**	**ITU**	**GIH**	**Disease**
rs77931234-G	*ACADM*	0.037	0.000	0.000	0.000	0.000	0.000	Acetyl-coA dehydrogenase deficiency (Rocha et al., 2014)
rs777176261-A	*BIN1*	0.012	0.000	0.000	0.000	0.000	0.000	Centronuclear myopathy (Cabrera-Serrano et al., 2018)
rs80338934-A	*SH3TC2*	0.006	0.000	0.000	0.000	0.000	0.000	Charcot–Marie–Tooth disease (Claramunt et al., 2007; Sevilla et al., 2013)
rs119483085-A	*NDRG1*	0.006	0.000	0.000	0.000	0.000	0.000	Charcot–Marie–Tooth disease (Claramunt et al., 2007; Sevilla et al., 2013)
rs1801968-G	*TOR1A*	0.019	0.1	0.141	0.157	0.165	0.211	Dystonia (Kalaydjieva et al., 2001)
rs1126809-A	*TYR*	0.154	0.305	0.269	0.079	0.006	0.103	Oculocutaneous albinism (Gamella et al., 2013)
rs104894396-T	*GJB2*	0.019	0.000	0.000	0.007	0.000	0.000	Deafness (Álvarez et al., 2005; Bouwer et al., 2007)

*Variant rs ID and risk allele, risk allele frequency for each population and disease association are shown.*

We next investigated the 334 pathogenic variants described in the ClinVar database present in our dataset. In Roma, we found 60 out of 334 variants segregating at low RAF in the population. Only 27 variants have a RAF difference equal or higher than 1.5 comparing Roma and non-Roma (Supplementary Table 8). Although the RAF of these variants is low (below 5% in most cases), there are variants with increased frequency in Roma, but interestingly, we observe disease-associated variants with increased frequencies in European and South Asian non-Roma populations. For example, rs1799807 (chr 3:165548529) is a missense pathogenic variant causing the deficiency of butyrylcholine esterase and, consequently, postanesthetic apnea (McGuire et al., 1989; Jasiecki et al., 2019). This variant is only present in European populations and virtually absent in South Asia and Roma (Supplementary Table 8). On the contrary, rs137941190 (chr 11:126215441) is a missense pathogenic variant for Al-Raqad syndrome, described in Pakistani patients (Ahmed et al., 2014). The risk allele of this variant is absent in the European exomes, but it appears at low frequencies in the Roma and South Asian samples (Supplementary Table 8), although the genotype frequencies are not statistically different (Supplementary Table 9). Regarding the ancestry inference, European ancestry is assigned for the risk allele of most of these variants (Supplementary Table 10), except for rs104894396 (chr 13:20763650), as explained above.

The screening of known disease-associated variants together with the local ancestry inference has allowed to report both the presence and the absence of particular mutations in the Roma and to trace their most likely ancestral origin. However, most known clinically relevant variants have been discovered in European populations, which leads to an ascertainment bias that can weaken the results when studying the Roma population: out of the 334 pathogenic variants, less than 45 variants are segregating in South Asian populations (43 in PJL, 41 in GIH, and 31 in ITU), while there are 84 and 74 segregating in IBS and TSI, respectively.

### Many Drug-Response Variants in Roma Are Related to Metabolic and Cardiovascular Disorders

Besides disease-associated variants, other functionally relevant mutations (e.g., pharmacogenomic variants) exhibit inter-population genetic variation in the human genome, as mentioned above. Regarding drug binding domains, we identified 101 variants in our dataset that disrupt the domains without being deleterious for the protein. This set is less biased toward European genetic variation, since it is based on impact prediction, rather than on previously discovered genetic associations (Dopazo et al., 2016). Only 26 variants were found to have a MAF fold increase ≥1.5 comparing Roma and non-Roma (Supplementary Table 11). Variants with known association drug phenotypes reported in PharmGKB (Whirl-Carrillo et al., 2012) have higher MAF in European populations, showing the European-centric bias in biomedical genetic studies. For example, the rs5918 variant, located in the *ITGB3* gene (chr17:45360730), reduces the efficacy of aspirin and clopidogrel (Dropinski et al., 2007; Motovska et al., 2009) (indicated for coronary artery disease and myocardial infarction) and it shows a higher allele frequency in IBS and TSI than in Roma (Supplementary Table 11). A variant found in the *GLP1R* gene (rs6923761; chr6:39034072) reduces the treatment efficacy for obesity and type II diabetes (i.e., sitagliptin, vildagliptin, and liraglutide) (Javorský et al., 2016) and its frequency is significantly lower in Roma than in the tested European populations (Supplementary Table 11).

On the contrary, there are 13 variants with increased MAF in Roma (Supplementary Table 11) with significantly different genotype frequencies compared to non-Roma groups (Supplementary Table 12). For example, there is a variant in the *CRYZ* gene (chr1:75175886) with significantly higher MAF in Roma and South Asians than in Europeans (Supplementary Table 11). Its protein contains a drug binding domain for dicumarol, indicated for deep vein thrombosis (Supplementary Table 11) and 19 out of 27 minor alleles in Roma have South Asian ancestry (Supplementary Table 13). In the *PTPRE* gene drug binding domain for alendronate (indicated for osteoporosis) (Supplementary Table 11), we identify a variant (chr10:129868686) in Roma (3.7%), virtually absent in non-Roma, except GIH (0.5%) (Supplementary Table 11). Four Roma individuals were heterozygotes for this variant: one with both haplotypes assigned to South Asian ancestry and three with one European and one South Asian haplotype (Supplementary Table 13), which suggests that this variant in the Roma originated in South Asia. Regarding the ancestry inference and besides the mentioned examples, many of the variants have the minor allele with European ancestry: 72% of the minor alleles are assigned to a European-related ancestry; slightly above the mean genome-wide ancestry (68.4%), but within the first SD of the distribution. Although experimental evidence suggesting these variants affect the binding of these drugs is lacking, follow-up studies should be performed to validate the functional impact of these variants.

We next examined previously described variants in ADME genes. In our dataset, 14 out of 95 of them show increased MAF with a fold change equal or higher than 1.5 comparing Roma and non-Roma (Table 3). Some variants found in European groups are absent in the Roma exomes: e.g., rs34130495, which modifies the metabolism of tramadol (indicated for mild-to-moderate pain) (Table 3). However, many of the variants with increased frequencies in Spanish Roma are clinically associated with metabolic and cardiovascular-related drugs (Table 3) and some have significantly different genotype frequencies between Roma and non-Roma (Supplementary Table 14). For example, the rs4149056 variant shows a higher MAF in Roma than in IBS (18% and 11%, respectively) (Table 3) and it increases the risk of toxicity to simvastatin (indicated for hypercholesterolemia) (Table 3). A previous study reports a frequency of 17.2 and 18.9% of this variant in Roma and non-Roma groups from Hungary, respectively (Nagy et al., 2015), suggesting that this variant in the Spanish Roma was present before the arrival into the Iberian Peninsula. rs316019 variant also shows a significant MAF increase in Roma than in IBS and TSI populations (20%, 9%, and 11%, respectively) (Table 3) and it is reported to modify the metabolism of metformin, a drug used to treat type II diabetes (Table 3). Regarding ancestry inference, the minor alleles of these variants are almost exclusively of European ancestry (Supplementary Table 15). Lastly, three previously found variants with increased frequencies in Croatian Roma (Škarić-Jurić et al., 2018) do not show significantly higher MAFs in Spanish Roma (rs10509681, rs8192709, and rs34059508) (Table 3).

**TABLE 3:** List of variants in ADME genes found to have a fold increase in allele frequency equal or higher than 1.5 comparing Roma and non-Roma.

**Variant – allele**	**Gene**	**Roma**	**IBS**	**TSI**	**PJL**	**ITU**	**GIH**	**Clinical annotation**
rs12208357-T	*SLC22A1*	0.019 (0.022)	0.058	0.058	0.021	0.024	0.010	Metabolism metformin (Shu et al., 2008; Sundelin et al., 2017)
rs2282143-T	*SLC22A1*	0.037 (0.085)	0.005	0.013	0.079	0.059	0.082	Metabolism metformin (Yoon et al., 2013)
rs34130495-A	*SLC22A1*	0.000 (0.023)	0.032	0.019	0.000	0.000	0.000	Metabolism tramadol (Tzvetkov et al., 2011)
rs34059508-A	*SLC22A1*	0.012 (**0.048**)	0.021	0.006	0.000	0.000	0.000	Metabolism metformin (Shu et al., 2008)
rs316019-A	*SLC22A2*	0.204 (0.066)	0.089	0.109	0.107	0.165	0.134	Metabolism metformin; Toxicity cisplatin-anthracyclines (Visscher et al., 2012; Yoon et al., 2013)
rs1800460-T	*TPMT*	0.025 (0.002)	0.037	0.019	0.000	0.000	0.000	Toxicity azathioprine and mercaptopurine (Stocco et al., 2012; Steponaitiene et al., 2016)
rs717620-T	*ABCC2*	0.185 (0.253)	0.211	0.186	0.071	0.047	0.077	Efficacy and dosage atorvastatin; Toxicity fluorouracil (Cecchin et al., 2013; Prado et al., 2018)
rs4244285-A	*CYP2C19*	0.191 (0.155)	0.133	0.090	0.314	0.388	0.330	Efficacy and toxicity clopidogrel; Efficacy amitriptyline
rs10509681-C	*CYP2C8*	0.099 (**0.165**)	0.168	0.141	0.050	0.024	0.041	Metabolism rosiglitazone (Dawed et al., 2016)
rs1058930-C	*CYP2C8*	0.105 (0.056)	0.042	0.051	0.014	0.006	0.010	Metabolism diclofenac (Dorado et al., 2008)
rs4149056-C	*SLCO1B1*	0.179 (0.102)	0.111	0.224	0.050	0.076	0.021	Toxicity simvastatin (Shek et al., 2017)
rs1048943-C	*CYP1A1*	0.037 (0.033)	0.016	0.045	0.107	0.118	0.113	Efficacy capecitabine and docetaxel (Dong et al., 2012)
rs1799814-T	*CYP1A1*	0.043 (0.033)	0.089	0.013	0.014	0.018	0.005	Metabolism dacarbazine (Lewis et al., 2016)
rs8192709-T	*CYP2B6*	0.093 (**0.128**)	0.047	0.064	0.043	0.035	0.036	Toxicity efavirenz (Dhoro et al., 2014; Dickinson et al., 2016)

*Variant rs ID and risk allele, gene, risk allele frequency for each population and PharmGKB main clinical annotations are shown. Croatian Roma frequencies from Škarić-Jurić et al., 2018 are also included within brackets in the Roma column (those variants with significantly higher frequencies are in bold).*

The screening beyond disease-associated variants in the Roma population reveals that most of them can change the response of drugs used for metabolic and cardiovascular disorders and that they might have a European origin. However, this analysis is based on the impact prediction and it is important to take into account that the phenotype is also influenced by the environment, non-coding variants and regulatory elements, among others.

## Discussion

The underrepresentation of human populations in genetic studies impairs the understanding of genome architecture and exacerbates health differences. In order to overcome this limitation, the Eurocentric bias in the discovery of functional variants has to be taken into account (Need and Goldstein, 2009; Popejoy and Fullerton, 2016; Bentley et al., 2017; Martin et al., 2019; Sirugo et al., 2019). In the case of the Roma population, we found that the genetic portability with European populations is overall high: allele sharing and LD decay patterns are comparable among groups. However, low frequency variants (MAF < 5%) can present some challenges in the genetic characterization of this population, since only half of these variants in the Roma are also segregating in non-Roma populations. This is consistent with the fact that low frequency and rare variants are more population structured than common variants (Casals and Bertranpetit, 2012; Bomba et al., 2017).

The overrepresentation analysis shows enrichment of some gene sets with increased MAF deleterious variants for genetic disorders. Interestingly, this enrichment occurs both in Roma and in non-Roma, consistent with previous literature. For example, we identify an enrichment for non-triple negative breast cancer in non-Roma, in agreement with a lower incidence in Roma, which show more triple negative cases (Reckova et al., 2017). *Triple negative* refers to the overexpression of three common markers (i.e., estrogen receptor, progesterone receptor or *HER2* oncogene) (Foulkes et al., 2010). However, the overexpression of these markers is common in breast cancer patients of European descent, but not in other groups, such as African-descent (Brewster et al., 2014) or Roma (Reckova et al., 2017) patients.

In the present study, we also examine previously defined disease-associated variants. Besides confirming the ancestry origin of some Mendelian mutations in the Roma, we provide new evidence: the risk allele responsible for Acetyl-coA dehydrogenase deficiency (rs77931234) is traced to a South Asian-like ancestry, while the risk alleles for Charcot–Marie–Tooth disease variants (rs119483085, rs80338934) show European-related ancestry. In addition, we perform a comprehensive study of the pathogenic variation reported in ClinVar database (Landrum et al., 2014). The results show a different frequency spectrum of previously identified variants when comparing Roma and non-Roma groups. However, most of the variants in the Roma are traced to a European origin, evidencing the Eurocentric bias in public databases (Kessler et al., 2016).

Regarding pharmacogenetic variation, we identify variants with increased and decreased MAF in Roma. Many of the variants in drug-binding domains that we found with increased frequencies in non-Roma European populations have been previously associated with a drug response trait, while those with increased frequencies in Roma or South Asian groups are still not specifically characterized. This is particularly relevant, since many of them are related to metabolic and cardiovascular drugs and previous studies suggest that Roma show higher prevalence of these diseases (Vozarova De Courten et al., 2003; Živković et al., 2010). Following the expectations of European ancestry proportions, most drug-response variants in Roma are traced to a European-related origin, which is not a signal of bias, because the list of variants that disrupt drug binding domains is not based on previously known associations discovered in populations of European descent (Dopazo et al., 2016). Thus, these variants show inter-population variability in the Roma, since a differential European gene flow among Roma groups has been previously described (Mendizabal et al., 2012; Font-Porterias et al., 2019). In this sense, the variants reported with increased frequency in Spanish Roma do not completely overlap with a similar study in Croatian Roma (Škarić-Jurić et al., 2018). Moreover, the significantly increased frequency of a *CYP2C19* polymorphism (rs4244285) in Hungarian and Portuguese Roma groups (Sipeky et al., 2013; Teixeira et al., 2015) is not observed in Spanish Roma.

For complex diseases, polygenic risk scores (PRSs) are designed to predict the phenotype from genetic data, combining the effect sizes of multiple variants and their frequency (Wray et al., 2007). However, the pre-computed effect sizes usually derive from genome-wide association studies (GWAS), and given its Eurocentric bias, PRSs have a greater predictive accuracy in populations with European ancestry (Martin et al., 2017, 2019; De La Vega and Bustamante, 2018; Kim et al., 2018; Gurdasani et al., 2019). Particularly, they show a systematic bias when applied to other populations due to several factors: (i) GWAS are biased toward those variants segregating in the study population; (ii) when two populations have different LD patterns, tagSNPs and causal variants can differ; and (iii) environmental and genetic factors can be confounded when phenotypes are geographically stratified (Martin et al., 2019; Sirugo et al., 2019). Although new methods are emerging to overcome these limitations (Márquez-Luna et al., 2017; Marnetto et al., 2020), an in-depth analysis of PRS accuracy in non-European-descent populations is needed before implementing them to other under-represented populations (Sirugo et al., 2019).

Here, we provide new evidence of a different frequency spectrum of clinically relevant variants across populations: while some have increased allele frequencies in the Roma, others are virtually absent. This was possible due to the availability of a substantial number of whole-exome sequences at high coverage, which allows the study of clinically relevant genetic variation, enriched in low frequency variants (Bomba et al., 2017). However, this different frequency spectrum cannot be directly attributed to a lower prevalence of some diseases; instead, other unstudied variants might be responsible for disease susceptibility and drug response in this population. Although it is an exploratory approach without functional validation, this study aims to shift the traditional paradigm of focusing only on the increased genetic risk for some diseases in the so-called “isolated” populations. In fact, these results further confirm that Roma are not so genetically isolated. Gene flow with European groups accounts for 65% of their genetic ancestry (Font-Porterias et al., 2019); thus, clinically damaging variants are traced both to South Asian and European-related haplotypes. Lastly, we caution that these results are geographically limited to the Roma population from Spain, and further characterization should be performed in other groups with different demographic trajectories and with increasing sample sizes.

We would like to remark that this study does not aim to exacerbate the importance of inter-population variability, to justify health differences in minority ethnic groups, or to advocate for racialized medicine. In fact, genetic ancestry is not the only determinant of ethnicity or health, and social factors should be considered. Given that genetic diversity is a continuum, large scale genome-wide studies are needed to fully capture and represent human variation, without excluding any population while respecting their rights and interests and properly accounting for demographic differences. This would prevent the current overgeneralization of the results obtained from genetic studies on populations with only European ancestry in the assessment of disease risk testing and treatment response (Martin et al., 2019; Batai et al., 2020; Hudson et al., 2020).

## Data Availability Statement

The original contributions presented in the study are included in the article/Supplementary Material, further inquiries can be directed to the corresponding author/s.

## Ethics Statement

The studies involving human participants were reviewed and approved by the CEIC-Parc de Salut Mar 2019/8900/I. The patients/participants provided their written informed consent to participate in this study.

## Author Contributions

NF-P and DC contributed to the design and conception of the study. NF-P performed and implemented the data analysis. All authors contributed to the interpretation and discussion of the results and writing of the manuscript, and approved the submitted version.

## Conflict of Interest

The authors declare that the research was conducted in the absence of any commercial or financial relationships that could be construed as a potential conflict of interest.

## Footnotes

1 www.pharmaadme.org
