# Supplementary material for: Admixture Has Shaped Romani Genetic Diversity in Clinically Relevant Variants
Source: Front Genet. 2021 Jun 16;12:683880. doi: 10.3389/fgene.2021.683880 (PMC8244592; doi:10.3389/fgene.2021.683880)
Supplement: Supplementary file 1 [file Data_Sheet_1.DOCX]

***Supplementary material***

**Note S1. Glossary of terms**

Some terms used in genetic studies may have ambiguous meanings and are applied in different contexts (Caulfield et al. 2009; Ali-Khan et al. 2011; Peterson et al. 2019; Mathieson and Scally 2020). Thus, we include below the definitions used in the present study.

*Ethnicity:* construct to describe a group of people based on linguistic, cultural, social, historical factors, etc. Not all ethnic groups share similar ancestral origins; only some groups have common genetic factors. Here, the term ethnicity is not used as a synonym for ‘race’ or ‘racial categories’, which have politically charged meanings, often based on stereotypes, power dynamics and used to justify hierarchies and racism (Race Ethnicity and Genetics Working Group 2005; Cornell and Hartmann 2007; Mersha and Abebe 2015; Peterson et al. 2019).

*Ancestry:* description of the populations representing the series of ancestors reflected in the DNA of an individual. Here, ancestry is based on genetic similarities rather than in genealogical inference or cultural factors. However, we note that the use of discrete genetic ancestries underrepresents the actual complexity of genetic variation (Race Ethnicity and Genetics Working Group 2005; Peterson et al. 2019; Mathieson and Scally 2020).

*Population*: “group of individuals with shared genetic ancestry” (Peterson et al. 2019). Here, several non-Roma populations are included as a reference panel. These individuals are grouped according to the populations defined in their source publication: IBS, TSI, PJL, GIH and ITU (1000 Genomes Project Consortium 2012).

*Founder mutation:* mutation found at an increased frequency in a particular group of individuals (National Cancer Institute).

*Clinically-relevant variant:* functional genetic variant related to a benign or pathogenic clinical phenotype, including traits, diseases and treatment response differences. In turn, clinically-damaging variants are here defined as deleterious genetic mutations with a negative impact on a clinical outcome (Richards et al. 2008; Stanley et al. 2014; Richards et al. 2015).

**Note S2. Overrepresentation analysis to approximate gene mutation accumulation**

In order to compare the patterns of gene mutation accumulation among Roma and non-Roma groups, we examined the distributions of mean number of deleterious alleles (N_alleles_) and homozygotes (N_hom_) per individual per gene for GERP, PolyPhen and CADD deleterious categories. As expected, there is a high correlation between these distributions among Roma and non-Roma populations (Supplementary Fig. 2-3, Table S1-S2): both the genes that accumulate more deleterious alleles and the ones without them are shared across populations.

To further analyse the gene mutation accumulation, we performed an overrepresentation analysis, reporting that the most constrained genes (i.e. without any deleterious allele in any population) are significantly enriched in Gene Ontology (GO) terms related to essential pathways: axon development, cell morphogenesis, biosynthetic process, meiotic cell cycle etc. On the contrary, pseudogenes or non-coding genes are more prone to tolerate deleterious alleles.

**Note S3. Overrepresentation analysis for genes in OMIM traits**

To initially avoid the ascertainment bias and since Roma and non-Roma show differences in the frequency distribution of deleterious mutations, we performed a gene overrepresentation analysis of OMIM traits without relying on a pre-defined set of disease-associated variants. Particularly, we identified those genes with deleterious variants with a fold increase in MAF equal or higher than 5 in Roma or non-Roma, stratified in GERP, PolyPhen and CADD deleterious categories.

We selected those diseases to be enriched when the False Discovery Rate (FDR) adjusted p-value < 0.05 or when FDR > 0.05 but the uncorrected p-value < 0.05, the enrichment ratio > 45 and it was found in at least two variant annotation analyses (i.e. GERP, PolyPhen, CADD). After applying these criteria, the overrepresented disorders in Roma or non-Roma are classified in “Rare conditions”, “Cardiovascular and metabolic disorders” and “Other conditions” (Table 1, S3-S4).

The enrichment in OMIM traits with more evidence are fully explained in the Results section. Other disorders also fulfil the enrichment criteria, although the posterior curation does not strongly support the relationship between the overrepresentation results and an actual increased genetic risk. For example, tracheoesophageal fistula shows an overrepresentation of genes with higher frequency in non-Roma than in Roma (Table S3). However, Mendelian inheritance is unlikely and genetic factors do not appear to play a major role (Shaw-Smith 2006), thus there are no genetic variants described for this rare condition.

In addition, susceptibility to Parkinson shows an overrepresentation of genes with variants with higher frequency in non-Roma than in Roma (Table S3). The enrichment is specially noticed for South Asians against Roma consistent with a lower prevalence of Parkinson disease among Roma (Milanov et al. 2000). However, the susceptibility variant described in OMIM for ATXN2 gene is not available in our dataset, since it is a (CAG)n repeat expansion.

Lastly, in the Results section we describe a differential enrichment for the two types of diabetes but without the evidence of pathogenic or risk-factor variants. Regarding non-insulin dependent diabetes, one likely-benign variant in KCNJ11 gene associated with hyperinsulinemia (rs1800467) is virtually absent in Roma but is present in small frequencies in non-Roma populations (Table S4). For insulin dependent diabetes none of the pathogenic variants described in OMIM for OAS1 gene are available in our dataset or have allele frequency differences (Table S4).

**Supplementary Figures**

**
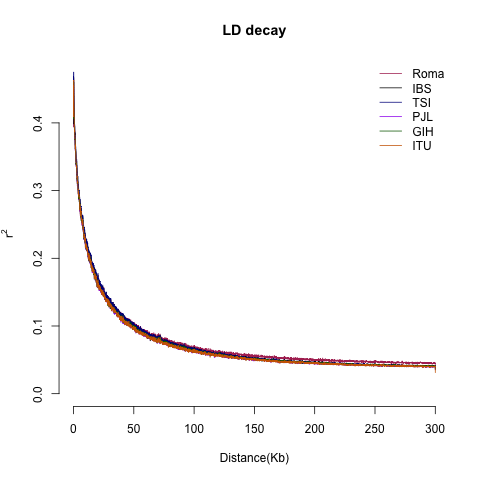
**

**Supplementary Figure 1.** Linkage disequilibrium decay: average correlation values (r^2^) across increasing genomic distances in kb for Roma and non-Roma populations. Data from genome-wide array dataset.

**
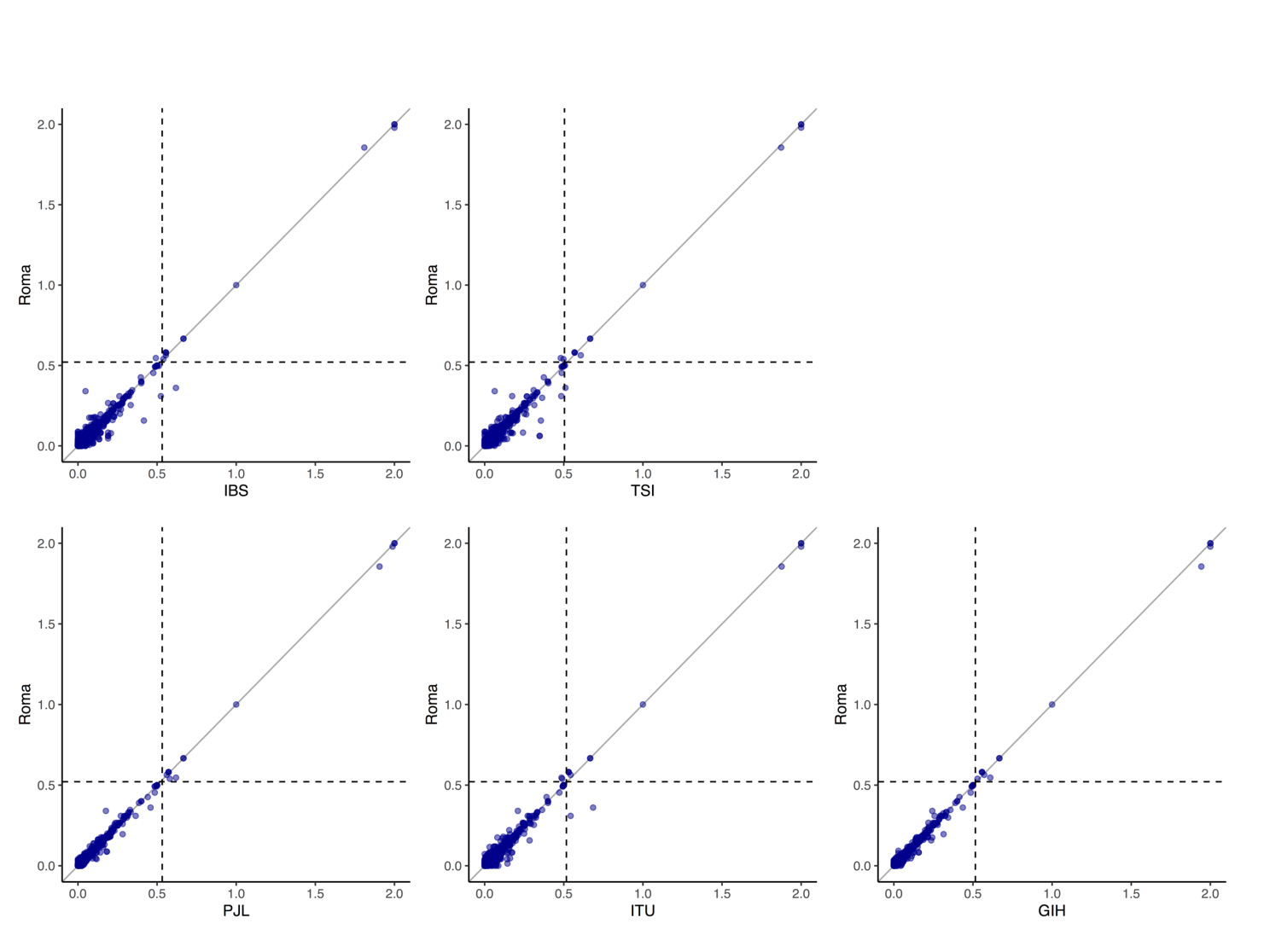
**

**Supplementary Figure 2.** Correlations between mean N_alleles_ per gene using the variants with 2 < GERP < 4 in Roma and non-Roma populations. N_alleles_ are normalized by number of variants in each gene (both axis range from 0 to 2). Each panel show a Roma vs non-Roma group comparison. Each dot represents a gene. Dashed lines are 99.9% percentiles in each population.


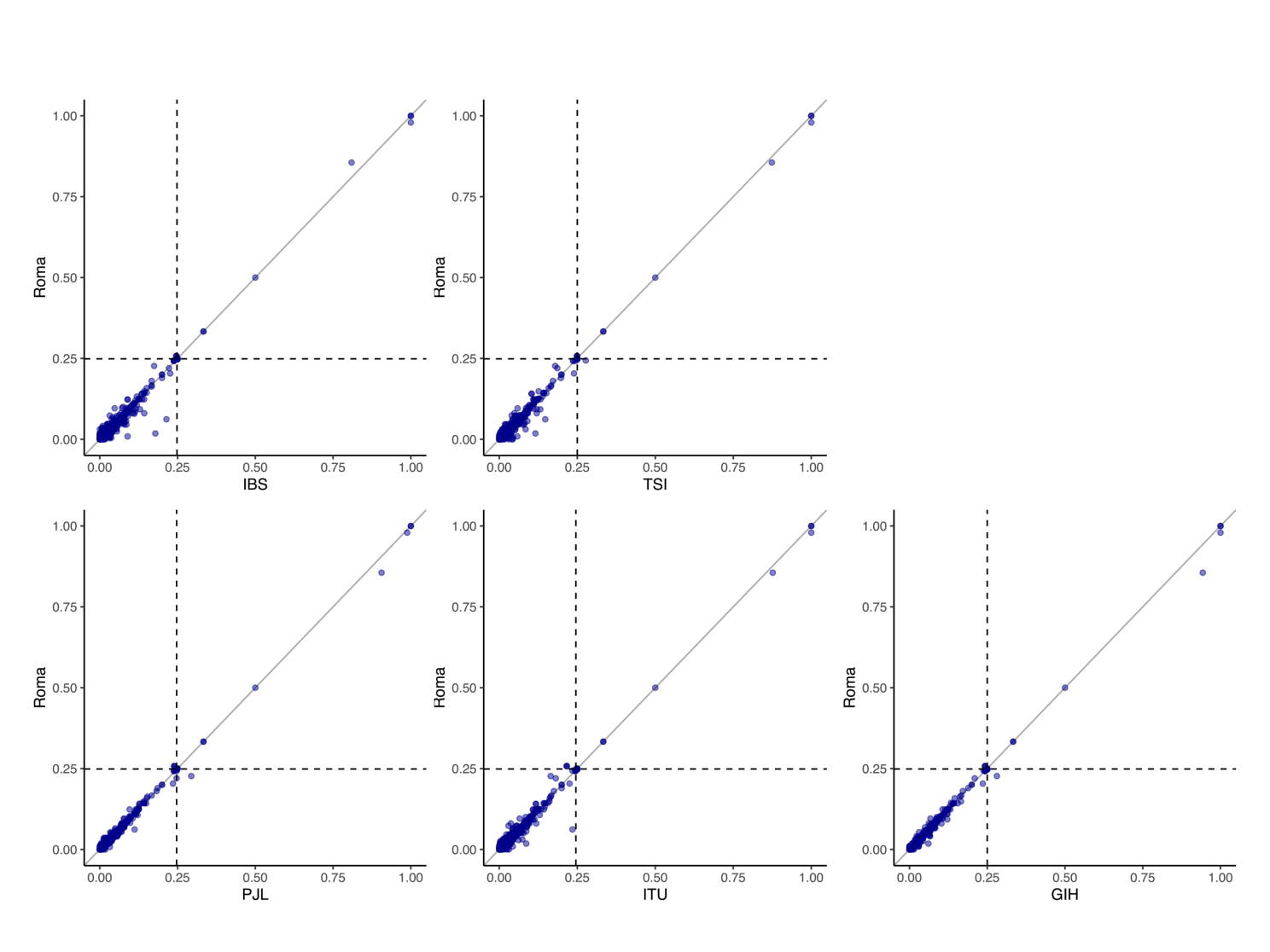


**Supplementary Figure 3.** Correlations between mean N_hom_ per gene using the variants with 2 < GERP < 4 in Roma and non-Roma populations. N_hom_ are normalized by number of variants in each gene (both axis range from 0 to 1). Each panel show a Roma vs non-Roma group comparison. Each dot represents a gene. Dashed lines are 99.9% percentiles in each population.

**Supplementary Tables**

|  | **N_alleles_ 2 < GERP < 4** | | **N_alleles_ 4 < GERP < 6** | | **N_alleles_ 6 < GERP** | | **N_alleles_ PolyP poss.del** | | **N_alleles_ PolyP prob.del** | | **N_alleles_ 10 < CADD < 20** | | **N_alleles_ 20 < CADD < 30** | | **N_alleles_ 30 < CADD** | |
| --- | --- | --- | --- | --- | --- | --- | --- | --- | --- | --- | --- | --- | --- | --- | --- | --- |
|  | **R^2^** | **Slope** | **R^2^** | **Slope** | **R^2^** | **Slope** | **R^2^** | **Slope** | **R^2^** | **Slope** | **R^2^** | **Slope** | **R^2^** | **Slope** | **R^2^** | **Slope** |
| **Roma/IBS** | 0.986 | 0.977 | 0.952 | 0.942 | 0.93 | 0.927 | 0.931 | 0.781 | 0.758 | 0.746 | 0.971 | 0.969 | 0.951 | 0.956 | 0.912 | 1.023 |
| **Roma/TSI** | 0.986 | 0.975 | 0.963 | 0.94 | 0.927 | 0.834 | 0.936 | 0.844 | 0.827 | 0.854 | 0.970 | 0.953 | 0.953 | 0.944 | 0.875 | 1.072 |
| **Roma/PJL** | 0.997 | 0.990 | 0.991 | 1.009 | 0.986 | 0.909 | 0.981 | 0.99 | 0.949 | 0.897 | 0.956 | 0.921 | 0.933 | 0.948 | 0.781 | 0.747 |
| **Roma/ITU** | 0.989 | 0.98 | 0.966 | 0.975 | 0.929 | 0.835 | 0.944 | 0.925 | 0.812 | 0.846 | 0.946 | 0.920 | 0.928 | 0.913 | 0.755 | 0.628 |
| **Roma/GIH** | 0.997 | 0.988 | 0.984 | 1.016 | 0.986 | 0.896 | 0.974 | 1.007 | 0.939 | 0.801 | 0.940 | 0.892 | 0.923 | 0.933 | 0.723 | 0.655 |

**Table S1.** Correlation between missense N_alleles_ per gene for each category in Roma compared to non-Roma populations. Linear regression was applied to calculate the slope of each distribution.

|  | **N_hom_ 2 < GERP < 4** | | **N_hom_ 4 < GERP < 6** | | **N_hom_ 6 < GERP** | | **N_hom_ PolyP poss.del** | | **N_hom_ PolyP prob.del** | | **N_hom_ 10 < CADD < 20** | | **N_hom_ 20 < CADD < 30** | | **N_hom_ 30 < CADD** | |
| --- | --- | --- | --- | --- | --- | --- | --- | --- | --- | --- | --- | --- | --- | --- | --- | --- |
|  | **R^2^** | **Slope** | **R^2^** | **Slope** | **R^2^** | **Slope** | **R^2^** | **Slope** | **R^2^** | **Slope** | **R^2^** | **Slope** | **R^2^** | **Slope** | **R^2^** | **Slope** |
| **Roma/IBS** | 0.99 | 0.984 | 0.968 | 0.956 | 0.909 | 1.093 | 0.915 | 0.763 | 0.831 | 0.693 | 0.953 | 0.929 | 0.940 | 0.963 | 0.730 | 0.973 |
| **Roma/TSI** | 0.993 | 0.985 | 0.974 | 0.964 | 0.958 | 0.902 | 0.919 | 0.799 | 0.839 | 0.751 | 0.963 | 0.904 | 0.941 | 0.992 | 0.469 | 0.985 |
| **Roma/PJL** | 0.998 | 0.988 | 0.987 | 1.001 | 0.993 | 0.884 | 0.987 | 0.938 | 0.95 | 0.892 | 0.948 | 0.848 | 0.938 | 0.932 | 0.793 | 0.653 |
| **Roma/ITU** | 0.993 | 0.984 | 0.971 | 0.988 | 0.961 | 0.834 | 0.949 | 0.955 | 0.879 | 0.834 | 0.932 | 0.874 | 0.930 | 0.883 | 0.814 | 0.453 |
| **Roma/GIH** | 0.998 | 0.98 | 0.986 | 1.007 | 0.988 | 0.901 | 0.980 | 0.985 | 0.89 | 0.848 | 0.933 | 0.848 | 0.9183 | 0.888 | 0.796 | 0.606 |

**Table S2.** Correlation between missense N_hom_ per gene for each category in Roma compared to non-Roma populations. Linear regression was applied to calculate the slope of each distribution

| **Description** | **Gene set size** | **Expect** | **Ratio** | **P Value** | **FDR** | **Comparison** | **Category** |
| --- | --- | --- | --- | --- | --- | --- | --- |
| Epidermolysis bullosa, junctional, non-Herlitz | 5 | 0.0209 | 95.857 | 0.0001 | 0.0084 | Roma-IBS | 4 > GERP < 6 |
| Epidermolysis bullosa, junctional, non-Herlitz | 5 | 0.013413 | 149.11 | 0.00001 | 0.0033 | Roma-IBS | PolyPhen prob. del |
| Epidermolysis bullosa, junctional, non-Herlitz | 5 | 0.014903 | 67.1 | 0.014823 | 0.23093 | Roma-IBS | 2 > GERP < 4 |
| Epidermolysis bullosa, junctional, non-Herlitz | 5 | 0.019374 | 51.615 | 0.019236 | 0.17442 | Roma-TSI | 4 > GERP < 6 |
| Epidermolysis bullosa, junctional, non-Herlitz | 5 | 0.019374 | 51.615 | 0.019236 | 0.17442 | Roma-TSI | 4 > GERP < 6 |
| Epidermolysis bullosa, junctional, non-Herlitz | 5 | 0.017884 | 55.917 | 0.017767 | 0.21014 | Roma-PJL | 4 > GERP < 6 |
| Epidermolysis bullosa, junctional, non-Herlitz | 5 | 0.019374 | 51.615 | 0.019236 | 0.15919 | Roma-GIH | 4 > GERP < 6 |
| Epidermolysis bullosa, junctional, non-Herlitz | 5 | 0.0074516 | 134.2 | 0.0074338 | 0.15453 | Roma-PJL | PolyPhen poss.del |
| Epidermolysis bullosa, junctional, non-Herlitz | 5 | 0.011923 | 83.875 | 0.011873 | 0.20580 | Roma-TSI | PolyPhen prob.del |
| Epidermolysis bullosa, junctional, non-Herlitz | 5 | 0.0089419 | 111.83 | 0.0089152 | 0.14802 | Roma-PJL | PolyPhen prob.del |
| Epidermolysis bullosa, junctional, non-Herlitz | 5 | 0.014903 | 67.1 | 0.014823 | 0.19270 | Roma-ITU | PolyPhen prob.del |
| Epidermolysis bullosa, junctional, non-Herlitz | 5 | 0.010432 | 95.857 | 0.010395 | 0.17251 | Roma-GIH | PolyPhen prob.del |
| Epidermolysis bullosa, junctional, non-Herlitz | 5 | 0.011923 | 83.875 | 0.011873 | 0.18502 | Roma-IBS | 10 < CADD < 20 |
| Epidermolysis bullosa, junctional, non-Herlitz | 5 | 0.013413 | 74.556 | 0.013349 | 0.16640 | Roma-PJL | 20 < CADD < 30 |
| Tetralogy of Fallot | 7 | 0.014605 | 136.94 | 0.000077992 | 0.0040556 | ITU-Roma | PolyPhen poss. del |
| Tetralogy of Fallot | 7 | 0.016692 | 59.911 | 0.016587 | 0.17251 | PJL-Roma | 2 > GERP < 4 |
| Tetralogy of Fallot | 7 | 0.018778 | 53.254 | 0.018644 | 0.24237 | ITU-Roma | 2 > GERP < 4 |
| Tetralogy of Fallot | 7 | 0.018778 | 53.254 | 0.018644 | 0.24237 | GIH-Roma | 2 > GERP < 4 |
| Tetralogy of Fallot | 7 | 0.018778 | 53.254 | 0.018644 | 0.18444 | ITU-Roma | 4 > GERP < 6 |
| Tetralogy of Fallot | 7 | 0.0041729 | 239.64 | 0.0041691 | 0.21680 | IBS-Roma | PolyPhen poss.del |
| Tetralogy of Fallot | 7 | 0.0062593 | 159.76 | 0.0062481 | 0.20077 | TSI-Roma | PolyPhen poss.del |
| Tetralogy of Fallot | 7 | 0.012519 | 79.881 | 0.012463 | 0.21602 | PJL-Roma | PolyPhen poss.del |
| Tetralogy of Fallot | 7 | 0.0062593 | 159.76 | 0.0062481 | 0.16654 | GIH-Roma | PolyPhen poss.del |
| Tetralogy of Fallot | 7 | 0.016692 | 59.911 | 0.016587 | 0.21564 | IBS-Roma | 20 < CADD < 30 |
| Tetralogy of Fallot | 7 | 0.014605 | 68.469 | 0.014527 | 0.27908 | TSI-Roma | 20 < CADD < 30 |
| Tetralogy of Fallot | 7 | 0.012519 | 79.881 | 0.012463 | 0.27547 | PJL-Roma | 20 < CADD < 30 |
| Tetralogy of Fallot | 7 | 0.020864 | 47.929 | 0.020697 | 0.20473 | ITU-Roma | 20 < CADD < 30 |
| Tetralogy of Fallot | 7 | 0.018778 | 53.254 | 0.018644 | 0.18953 | GIH-Roma | 20 < CADD < 30 |
| Tracheoesophageal fistula | 18 | 0.032191 | 62.130 | 0.00040274 | 0.018799 | PJL-Roma | PolyPhen poss. del |
| Tracheoesophageal fistula | 18 | 0.037556 | 53.254 | 0.00056204 | 0.014613 | ITU-Roma | PolyPhen poss. del |
| Tracheoesophageal fistula | 18 | 0.021461 | 93.194 | 0.00016212 | 0.0084305 | TSI-Roma | PolyPhen prob. del |
| Tracheoesophageal fistula | 18 | 0.032191 | 62.130 | 0.00040274 | 0.020942 | GIH-Roma | PolyPhen prob. del |
| Tracheoesophageal fistula | 18 | 0.037556 | 53.254 | 0.00056204 | 0.029226 | TSI-Roma | 20 < CADD < 30 |
| Tracheoesophageal fistula | 18 | 0.048286 | 41.420 | 0.00095739 | 0.049784 | GIH-Roma | 20 < CADD < 30 |
| Tracheoesophageal fistula | 18 | 0.016095 | 62.130 | 0.016014 | 0.36944 | IBS-Roma | 2 > GERP < 4 |
| Tracheoesophageal fistula | 18 | 0.016095 | 62.130 | 0.016014 | 0.16654 | GIH-Roma | PolyPhen poss.del |
| Tracheoesophageal fistula | 18 | 0.016095 | 62.130 | 0.016014 | 0.41636 | PJL-Roma | PolyPhen prob.del |
| Tracheoesophageal fistula | 18 | 0.0053651 | 186.39 | 0.0053651 | 0.27899 | TSI-Roma | 30 < CADD |
| Tracheoesophageal fistula | 18 | 0.010730 | 93.194 | 0.010703 | 0.27828 | ITU-Roma | 30 < CADD |
| Tracheoesophageal fistula | 18 | 0.0053651 | 186.39 | 0.0053651 | 0.27899 | GIH-Roma | 30 < CADD |
| Obesity-Leanness | 15 | 0.044709 | 44.733 | 0.00082260 | 0.042775 | Roma-IBS | 2 > GERP < 4 |
| Obesity-Leanness | 15 | 0.031297 | 63.905 | 0.00038687 | 0.020117 | Roma-TSI | 2 > GERP < 4 |
| Obesity-Leanness | 15 | 0.031297 | 63.905 | 0.00038687 | 0.020117 | Roma-PJL | 2 > GERP < 4 |
| Obesity-Leanness | 15 | 0.017884 | 55.917 | 0.017772 | 0.23104 | Roma-IBS | PolyPhen poss.del |
| Obesity-Leanness | 15 | 0.013413 | 74.556 | 0.013357 | 0.23152 | Roma-TSI | PolyPhen poss.del |
| Ischemic stroke | 5 | 0.010432 | 95.857 | 0.010395 | 0.21602 | Roma-PJL | 2 > GERP < 4 |
| Ischemic stroke | 5 | 0.016393 | 61 | 0.016296 | 0.20307 | Roma-GIH | 2 > GERP < 4 |
| Ischemic stroke | 5 | 0.020864 | 47.929 | 0.020703 | 0.17125 | Roma-IBS | 4 > GERP < 6 |
| Ischemic stroke | 5 | 0.019374 | 51.615 | 0.019236 | 0.17442 | Roma-TSI | 4 > GERP < 6 |
| Ischemic stroke | 5 | 0.017884 | 55.917 | 0.017767 | 0.21014 | Roma-PJL | 4 > GERP < 6 |
| Ischemic stroke | 5 | 0.019374 | 51.615 | 0.019236 | 0.15919 | Roma-GIH | 4 > GERP < 6 |
| Ischemic stroke | 5 | 0.013413 | 74.556 | 0.013349 | 0.22133 | Roma-IBS | PolyPhen prob.del |
| Ischemic stroke | 5 | 0.011923 | 83.875 | 0.011873 | 0.20580 | Roma-TSI | PolyPhen prob.del |
| Ischemic stroke | 5 | 0.0089419 | 111.83 | 0.0089152 | 0.14802 | Roma-PJL | PolyPhen prob.del |
| Ischemic stroke | 5 | 0.014903 | 67.1 | 0.014823 | 0.19270 | Roma-ITU | PolyPhen prob.del |
| Ischemic stroke | 5 | 0.010432 | 95.857 | 0.010395 | 0.17251 | Roma-GIH | PolyPhen prob.del |
| Ischemic stroke | 5 | 0.016393 | 61 | 0.016296 | 0.16872 | Roma-IBS | 20 < CADD < 30 |
| Ischemic stroke | 5 | 0.017884 | 55.917 | 0.017767 | 0.16115 | Roma-TSI | 20 < CADD < 30 |
| Ischemic stroke | 5 | 0.013413 | 74.556 | 0.013349 | 0.16640 | Roma-PJL | 20 < CADD < 30 |
| Ischemic stroke | 5 | 0.014903 | 67.1 | 0.014823 | 0.17548 | Roma-ITU | 20 < CADD < 30 |
| Ischemic stroke | 5 | 0.014903 | 67.1 | 0.014823 | 0.15354 | Roma-GIH | 20 < CADD < 30 |
| NonInsulin-dependent Diabetes Mellitus | 28 | 0.066766 | 44.933 | 0.00002837 | 0.0014751 | IBS-Roma | 4 > GERP < 6 |
| NonInsulin-dependent Diabetes Mellitus | 28 | 0.075112 | 39.940 | 0.00004231 | 0.0022003 | ITU-Roma | 4 > GERP < 6 |
| NonInsulin-dependent Diabetes Mellitus | 28 | 0.016692 | 59.911 | 0.016624 | 0.43223 | IBS-Roma | PolyPhen poss.del |
| Insulin-dependent Diabetes Mellitus | 5 | 0.020864 | 47.929 | 0.020703 | 0.17125 | Roma-IBS | 4 > GERP < 6 |
| Insulin-dependent Diabetes Mellitus | 5 | 0.017884 | 55.917 | 0.017767 | 0.21014 | Roma-PJL | 4 > GERP < 6 |
| Insulin-dependent Diabetes Mellitus | 5 | 0.019374 | 51.615 | 0.019236 | 0.15919 | Roma-GIH | 4 > GERP < 6 |
| Insulin-dependent Diabetes Mellitus | 5 | 0.0059613 | 167.75 | 0.0059506 | 0.18558 | Roma-IBS | PolyPhen poss.del |
| Insulin-dependent Diabetes Mellitus | 5 | 0.0044709 | 223.67 | 0.0044656 | 0.13929 | Roma-TSI | PolyPhen poss.del |
| Insulin-dependent Diabetes Mellitus | 5 | 0.0074516 | 134.2 | 0.0074338 | 0.15453 | Roma-PJL | PolyPhen poss.del |
| Insulin-dependent Diabetes Mellitus | 5 | 0.0089419 | 111.83 | 0.0089152 | 0.18502 | Roma-ITU | PolyPhen poss.del |
| Insulin-dependent Diabetes Mellitus | 5 | 0.0074516 | 134.2 | 0.0074338 | 0.15435 | Roma-GIH | PolyPhen poss.del |
| Insulin-dependent Diabetes Mellitus | 5 | 0.013413 | 74.556 | 0.013349 | 0.22133 | Roma-IBS | PolyPhen prob.del |
| Insulin-dependent Diabetes Mellitus | 5 | 0.011923 | 83.875 | 0.011873 | 0.20580 | Roma-TSI | PolyPhen prob.del |
| Insulin-dependent Diabetes Mellitus | 5 | 0.0089419 | 111.83 | 0.0089152 | 0.14802 | Roma-PJL | PolyPhen prob.del |
| Insulin-dependent Diabetes Mellitus | 5 | 0.014903 | 67.1 | 0.014823 | 0.19270 | Roma-ITU | PolyPhen prob.del |
| Insulin-dependent Diabetes Mellitus | 5 | 0.010432 | 95.857 | 0.010395 | 0.17251 | Roma-GIH | PolyPhen prob.del |
| Insulin-dependent Diabetes Mellitus | 5 | 0.016393 | 61 | 0.016296 | 0.16872 | Roma-IBS | 20 < CADD < 30 |
| Insulin-dependent Diabetes Mellitus | 5 | 0.017884 | 55.917 | 0.017767 | 0.16115 | Roma-TSI | 20 < CADD < 30 |
| Insulin-dependent Diabetes Mellitus | 5 | 0.013413 | 74.556 | 0.013349 | 0.16640 | Roma-PJL | 20 < CADD < 30 |
| Insulin-dependent Diabetes Mellitus | 5 | 0.014903 | 67.1 | 0.014823 | 0.17548 | Roma-ITU | 20 < CADD < 30 |
| Insulin-dependent Diabetes Mellitus | 5 | 0.014903 | 67.1 | 0.014823 | 0.15354 | Roma-GIH | 20 < CADD < 30 |
| Alcohol dependence | 6 | 0.017884 | 111.83 | 0.00011921 | 0.0061989 | Roma-ITU | 2 > GERP < 4 |
| Alcohol dependence | 6 | 0.019672 | 101.67 | 0.00014559 | 0.0075704 | Roma-GIH | 2 > GERP < 4 |
| Alcohol dependence | 6 | 0.017884 | 55.917 | 0.017764 | 0.23093 | Roma-IBS | 2 > GERP < 4 |
| Alcohol dependence | 6 | 0.012519 | 79.881 | 0.012463 | 0.25180 | Roma-TSI | 2 > GERP < 4 |
| Alcohol dependence | 6 | 0.012519 | 79.881 | 0.012463 | 0.21602 | Roma-PJL | 2 > GERP < 4 |
| Alcohol dependence | 6 | 0.0071535 | 139.79 | 0.0071375 | 0.18558 | Roma-IBS | PolyPhen poss.del |
| Alcohol dependence | 6 | 0.0053651 | 186.39 | 0.0053571 | 0.13929 | Roma-TSI | PolyPhen poss.del |
| Alcohol dependence | 6 | 0.0089419 | 111.83 | 0.0089152 | 0.15453 | Roma-PJL | PolyPhen poss.del |
| Alcohol dependence | 6 | 0.010730 | 93.194 | 0.010690 | 0.18502 | Roma-ITU | PolyPhen poss.del |
| Alcohol dependence | 6 | 0.0089419 | 111.83 | 0.0089152 | 0.15435 | Roma-GIH | PolyPhen poss.del |
| Alcohol dependence | 6 | 0.014307 | 69.896 | 0.014233 | 0.18502 | Roma-IBS | 10 < CADD < 20 |
| Alcohol dependence | 6 | 0.016095 | 62.130 | 0.016000 | 0.24237 | Roma-TSI | 10 < CADD < 20 |
| Alcohol dependence | 6 | 0.014307 | 69.896 | 0.014233 | 0.18502 | Roma-PJL | 10 < CADD < 20 |
| Alcohol dependence | 6 | 0.016095 | 62.130 | 0.016000 | 0.19390 | Roma-ITU | 10 < CADD < 20 |
| Alcohol dependence | 6 | 0.019672 | 50.833 | 0.019526 | 0.20307 | Roma-GIH | 10 < CADD < 20 |
| Breast cancer | 24 | 0.057228 | 34.948 | 0.0013379 | 0.034786 | PJL-Roma | 2 > GERP < 4 |
| Breast cancer | 24 | 0.042921 | 46.597 | 0.00072304 | 0.018799 | PJL-Roma | PolyPhen poss. del |
| Breast cancer | 24 | 0.071535 | 41.938 | 0.000037371 | 0.0019433 | PJL-Roma | 10 < CADD < 20 |
| Breast cancer | 24 | 0.021461 | 46.597 | 0.021314 | 0.36944 | IBS-Roma | 2 > GERP < 4 |
| Breast cancer | 24 | 0.021461 | 46.597 | 0.021314 | 0.18472 | GIH-Roma | PolyPhen poss.del |
| Parkinson disease, late-onset | 7 | 0.016692 | 59.911 | 0.016587 | 0.17251 | PJL-Roma | 2 > GERP < 4 |
| Parkinson disease, late-onset | 7 | 0.018778 | 53.254 | 0.018644 | 0.24237 | ITU-Roma | 2 > GERP < 4 |
| Parkinson disease, late-onset | 7 | 0.018778 | 53.254 | 0.018644 | 0.24237 | GIH-Roma | 2 > GERP < 4 |
| Parkinson disease, late-onset | 7 | 0.0062593 | 159.76 | 0.0062481 | 0.32490 | PJL-Roma | PolyPhen prob.del |
| Parkinson disease, late-onset | 7 | 0.012519 | 79.881 | 0.012463 | 0.30791 | ITU-Roma | PolyPhen prob.del |
| Parkinson disease, late-onset | 7 | 0.012519 | 79.881 | 0.012463 | 0.14802 | GIH-Roma | PolyPhen prob.del |
| Parkinson disease, late-onset | 7 | 0.020864 | 47.929 | 0.020697 | 0.21525 | PJL-Roma | 10 < CADD < 20 |
| Parkinson disease, late-onset | 7 | 0.020864 | 47.929 | 0.020697 | 0.20473 | GIH-Roma | 10 < CADD < 20 |

**Table S3.** List of enriched OMIM traits from WEB-based GEne SeT AnaLysis Toolkit (Liao et al. 2019), with OMIM term, gene set size (number of genes associated with each OMIM trait in the reference set), expected and observed enrichment ratios, uncorrected p-value, corrected p-value, population comparison (first population is the one showing increased allele frequency variants) and variant deleterious category (GERP, PolyPhen and CADD).

| **Variant** | **Allele** | **SA** | **EUR** | **UN** |
| --- | --- | --- | --- | --- |
| **rs2282440** | Minor | 25 | 0 | - |
|  | Major | 25 | 73 | 1 |
| **rs6025** | Minor | 1 | 7 | - |
|  | Major | 38 | 78 | - |
| **rs1229984** | Minor | 12 | 14 | 1 |
|  | Major | 21 | 74 | 2 |

**Table S6.** Local ancestry assignment (SA: South Asian, EUR: European, UN: Unassigned) for the minor and major alleles of the variants described in the gene enrichment analysis (Table S4). Minor allele matches the risk allele in all cases. Data from the merged WES-array dataset.

| **Variant - Allele** | **Position** | **Allele** | **SA** | **EUR** | **UN** |
| --- | --- | --- | --- | --- | --- |
| **rs1801968-G** | 9:132580901 | Minor | 1 | 2 | - |
|  |  | Major | 37 | 76 | 8 |
| **rs1126809-A** | 11:89017961 | Minor | 2 | 17 | 1 |
|  |  | Major | 32 | 67 | 5 |
| **Variant - Allele** | **Position** | **Genotype** | **SA-SA** | **EUR-EUR** | **EUR-SA** |
| **rs77931234-G** | 1:76226846 | Minor hom | - | - | - |
|  |  | Het | 1 | - | 3 |
| **rs777176261-A** | 2:127821221 | Minor hom | - | - | - |
|  |  | Het | - | - | 1 |
| **rs104894396-T** | 13:20763650 | Minor hom | - | - | - |
|  |  | Het | 1 | - | 3 |
| **rs119483085-A** | 8:134270617 | Minor hom | - | - | - |
|  |  | Het | - | 1 | - |
| **rs80338934-A** | 5:148389835 | Minor hom | - | - | - |
|  |  | Het | - | 1 | - |

**Table S7.** Local ancestry assignment (SA: South Asian, EUR: European, UN: Unassigned) for the minor and major alleles of the variants described in Table 2. Minor allele matches the risk allele in all cases. When the variant was filtered out with MAF<1% filter, only haplotype ancestry information could be retrieved (Minor hom: Minor homozygote; Het: Heterozygote). Data from the merged WES-array dataset.

| **Position - Allele** | | **Roma** | **IBS** | **TSI** | **PJL** | **GIH** | **ITU** | **Disease** |
| --- | --- | --- | --- | --- | --- | --- | --- | --- |
| 1:159175494 | T | 0.0185 | 0.0158 | 0.0192 | 0.0214 | 0 | 0 | Duffy blood group system  FY(bwk) phenotype |
| 1:17597423 | T | 0.0185 | 0.0105 | 0.0128 | 0 | 0.0103 | 0 | Uncombable hair syndrome |
| 1:45796892 | A | 0 | 0 | 0 | 0 | 0.0412 | 0 | Hereditary cancer-predisposing |
| 1:45797228 | T | 0 | 0.0215 | 0.013 | 0 | 0 | 0 | Endometrial cancer |
| 1:94473807 | T | 0.0309 | 0 | 0.0128 | 0.0071 | 0 | 0.0117 | Macular distrophy |
| 10:31810782 | C | 0.0185 | 0.0105 | 0 | 0 | 0 | 0 | Corneal dystrophy |
| 11:116691634 | A | 0.0247 | 0.0895 | 0.0705 | 0.0071 | 0.0052 | 0.0294 | Apolipoprotein A-IV polymorphism |
| 11:126215441 | T | 0.0062 | 0 | 0 | 0.0214 | 0.0103 | 0.0235 | Al-Raqad syndrome |
| 11:6645369 | T | 0.0370 | 0 | 0 | 0 | 0 | 0 | Mitral_valve_prolapse_2 |
| 12:121177150 | T | 0.0185 | 0 | 0 | 0 | 0 | 0 | Deficiency butyryl-CoA dehydrogenase |
| 12:7842655 | G | 0 | 0 | 0 | 0 | 0.0206 | 0.0059 | Microphthalmia, isolated 7 |
| 13:20763620 | G | 0 | 0.0215 | 0.0128 | 0 | 0 | 0 | Deafness |
| 13:20763650 | T | 0.0185 | 0 | 0 | 0.0071 | 0 | 0 | Hearing_impairment |
| 14:94844947 | T | 0.0062 | 0.0215 | 0 | 0 | 0 | 0 | Alpha-1-antitrypsin deficiency,  Chronic obstructive pulmonary disease |
| 17:56356502 | G | 0.0556 | 0.0268 | 0.021 | 0 | 0 | 0 | Myeloperoxidase deficiency |
| 19:33355167 | T | 0 | 0 | 0.0192 | 0 | 0 | 0 | Cystinuria |
| 3:15686693 | C | 0.0062 | 0.0263 | 0.0321 | 0.05 | 0.0309 | 0.0352 | Biotinidase deficiency |
| 3:165548529 | C | 0 | 0.0316 | 0.0321 | 0 | 0 | 0 | Butyrylcholine esterase deficiency |
| 4:187206919 | A | 0 | 0 | 0 | 0 | 0.0155 | 0 | Hereditary factor XI deficiency disease |
| 5:1216900 | A | 0.1235 | 0.2368 | 0.2532 | 0.1 | 0.1134 | 0.1176 | Iminoglycinuria, [Hyperglycinuria](https://www.omim.org/entry/138500) |
| 5:149921213 | A | 0.0309 | 0 | 0 | 0 | 0 | 0 | Mental retardation autosomal recessive |
| 5:150723155 | A | 0.0123 | 0.0105 | 0.0321 | 0.0071 | 0 | 0 | Iminoglycinuria, [Hyperglycinuria](https://www.omim.org/entry/138500) |
| 5:35072712 | G | 0 | 0.0211 | 0.0449 | 0 | 0 | 0 | Multiple fibroadenomas of the breast |
| 6:46679232 | T | 0 | 0 | 0 | 0.0286 | 0 | 0.0176 | Platelet-activating factor acetylhydrolase def. |
| 7:139715531 | A | 0 | 0 | 0 | 0 | 0.0155 | 0 | Ghosal hematodiaphyseal syndrome |
| 8:110100133 | C | 0.0370 | 0 | 0 | 0 | 0 | 0 | Hypothyroidism congenital nongoitrous |
| 9:104189856 | G | 0 | 0.0158 | 0.0128 | 0 | 0 | 0 | Hereditary fructosuria |

**Table S8.** List of ClinVar pathogenic variants found to have a fold increase in allele frequency equal or higher than 1.5 comparing Roma and non-Roma. Genomic position, risk allele, RAF for each population and disease association are shown.

| **Variant** | **Allele** | **SA** | **EUR** | **UN** |
| --- | --- | --- | --- | --- |
| **11:116691634** | Minor | - | 2 | - |
|  | Major | 23 | 99 | - |
| **5:1216900** | Minor | 5 | 10 | - |
|  | Major | 37 | 72 | - |
| **17:56356502** | Minor | - | 9 | - |
|  | Major | 48 | 67 | - |
| **3:15686693** | Minor | - | 1 | - |
|  | Major | 35 | 88 | - |

| **Variant** | **Genotype** | **SA-SA** | **EUR-EUR** | **EUR-SA** |
| --- | --- | --- | --- | --- |
| **11:126215441** | Minor hom | - | - | - |
|  | Het | - | - | 1 |
| **13:20763650** | Minor hom | - | - | - |
|  | Het | 1 | - | 2 |
| **1:94473807** | Minor hom | - | - | - |
|  | Het | - | - | 3 |
| **1:17597423** | Minor hom | - | - | - |
|  | Het | - | - | 2 |
| **10:31810782** | Minor hom | - | - | - |
|  | Het | - | 2 | - |
| **1:159175494** | Minor hom | - | - | - |
|  | Het | - | 2 | - |
| **5:150723155** | Minor hom | - | - | - |
|  | Het | - | 1 | - |
| **8:110100133** | Minor hom | - | 1 | - |
|  | Het | - | 3 | - |
| **5:149921213** | Minor hom | - | - | - |
|  | Het | 1 | - | 3 |
| **11:6645369** | Minor hom | - | - | - |
|  | Het | - | - | 4 |
| **12:121177150** | Minor hom | - | - | - |
|  | Het | 1 | - | 1 |

**Table S10.** Local ancestry assignment (SA: South Asian, EUR: European, UN: Unassigned) for the minor and major alleles of the variants described in Table S8. Minor allele matches the risk allele in all cases. When the variant was filtered out with MAF<1% filter, only haplotype ancestry information could be retrieved (Minor hom: Minor homozygote; Het: Heterozygote). Data from the merged WES-array dataset.

| **Position - Allele** | **Roma** | **IBS** | **TSI** | **PJL** | **ITU** | **GIH** | **Gene** | **Drug binding to domain affected by variant** | **Indication** |
| --- | --- | --- | --- | --- | --- | --- | --- | --- | --- |
| 1:20246876–T | 0.037 | 0.0737 | 0.0769 | 0.0929 | 0.1059 | 0.067 | PLA2G2E | Aminosalicylic Acid | Tuberculosis |
| 1:53679229 –G | 0.1049 | 0.1895 | 0.2564 | 0.0714 | 0.0529 | 0.0979 | CPT2 | L-Carnitine, Perhexiline | Carnitine deficiency, angina |
| 1:75175886-C | 0.2346 | 0.1211 | 0.1346 | 0.4071 | 0.4412 | 0.4742 | CRYZ | Dicumarol | Deep vein thrombosis |
| 2:198949344-G | 0.0000 | 0.0368 | 0.0321 | 0.0071 | 0.0000 | 0.0000 | PLCL1 | Quinacrine |  |
| 3:8809222-T | 0.2222 | 0.1222 | 0.1027 | 0.1029 | 0.1627 | 0.1383 | OXTR | Carbetocin, Oxytocin | Postpartum hemorrhage |
| 5:148206885-T (*) | 0.0000 | 0.0316 | 0.0128 | 0.0071 | 0.0000 | 0.0000 | ADRB2 | Acebutolol, Alprenolol, Arbutamine, Arformoterol, Asenapine, Bambuterol, etc. | Hypertension, chronic bronchitis, schizophrenia, etc. |
| 5:40691893-A | 0.0000 | 0.0158 | 0.0256 | 0.0071 | 0.0059 | 0.0000 | PTGER4 | Dinoprostone, Misoprostol | Labour induction, osteoarthritis, post-partum hemorrhage, etc. |
| 6:102516260-A | 0.0247 | 0.0158 | 0.0192 | 0.0000 | 0.0000 | 0.0000 | GRIK2 | Amobarbital, Aprobarbital, Butabarbital, Butalbital, Butethal, Heptabarbital, Hexobarbital, L-Glutamic | Insomnia, anxiety, headache |
| 6:39034072-A (*) | 0.1296 | 0.3632 | 0.2692 | 0.1714 | 0.1118 | 0.1082 | GLP1R | Exenatide, Glucagon recombinant, Liraglutide | Type 2 diabetes |
| 7:139715645-A | 0.0123 | 0.0158 | 0.0064 | 0.0071 | 0.0000 | 0.0052 | TBXAS1 | Ridogrel | Acute myocardial infarction |
| 8:143961102-T | 0.0741 | 0.0368 | 0,0000 | 0.1857 | 0.1706 | 0.1289 | CYP11B1 | Cimetidine, Clotrimazole, Etomidate, Fluconazole, Hydrocortisone, Ketoconazol, etc. | acid-reflux disorders, dermal infections, anesthesia, fungal infections, etc. |
| 9:35679251-G | 0.0864 | 0.0895 | 0.1154 | 0.05 | 0.0294 | 0.0309 | CA9 | Benzthiazide, Hydrochlorothiazide, Hydroflumethiazide, Zonisamide | Edema, hypertension, epilepsy |
| 10:101595996-A (*) | 0.0309 | 0.0895 | 0.1026 | 0.0071 | 0.0059 | 0.0155 | ABCC2 | Adenosine triphosphate, Arsenic trioxide, Atorvastatin, Carbamazepine, etc. | Acute promyelocytic leukemia, Hyperlipidemia, epilepsy, etc |
| 10:129868686-G | 0.0370 | 0.0000 | 0.0000 | 0.0000 | 0.0000 | 0.0052 | PTPRE | Alendronate | Osteoporosis |
| 10:5043821-T | 0.0000 | 0.0474 | 0.0192 | 0.0143 | 0.0471 | 0.0206 | AKR1C2 | NADH, Ursodeoxycholic acid | Primary Biliary Cholangitis, gallstone formation |
| 11:20648364-C | 0.0802 | 0.1474 | 0.0897 | 0.1857 | 0.1941 | 0.1289 | SLC6A5 | Glycine | Deep vein thrombosis, myocardial infarction, stroke, vascular occlusion |
| 11:92715117-G | 0.0247 | 0.0263 | 0.0192 | 0.0000 | 0.0118 | 0.0103 | MTNR1B | Agomelatine, Melatonin, Ramelteon | Depression, insomnia |
| 13:103718308-T | 0.0556 | 0.0632 | 0.0192 | 0.0000 | 0.0000 | 0.0000 | SLC10A2 | Acyclovir, Cyclosporine, Ursodeoxycholic acid, Valaciclovir | Immunosuppressive |
| 13:78475230-T | 0.0370 | 0.0263 | 0.0064 | 0.0000 | 0.0000 | 0.0000 | EDNRB | Bosentan, Sitaxentan | Pulmonary arterial hypertension |
| 14:95053863-T | 0.0988 | 0.0579 | 0.0577 | 0.0786 | 0.1 | 0.1134 | SERPINA5 | Drotrecogin alfa, Urokinase | Sepsis, embolism, coronary thrombosis |
| 14:95054012-G | 0.0988 | 0.0579 | 0.0577 | 0.0725 | 0.0976 | 0.1082 | SERPINA5 | Drotrecogin alfa, Urokinase | Sepsis, embolism, thrombosis |
| 15:75012985-C (*) | 0.037 | 0.0158 | 0.0449 | 0.1071 | 0.1176 | 0.1134 | CYP1A1 | Acetaminophen, Albendazole, Amiodarone, Amlodipine, Amodiaquine, Arsenic trioxide, Azelastine, etc. | Mild-moderate pain, neurocysticercosis, tachycardia, hypertension, malaria, etc. |
| 15:75012987-T | 0.0432 | 0.0895 | 0.0128 | 0.0143 | 0.0176 | 0.0052 | CYP1A1 | Acetaminophen, Albendazole, Amiodarone, Amlodipine, Amodiaquine, Arsenic trioxide, Azelastine, etc. | Mild-moderate pain, neurocysticercosis, tachycardia, hypertension, malaria, etc. |
| 16:1129193-T | 0.0741 | 0.0591 | 0.0395 | 0.1357 | 0.1412 | 0.1804 | SSTR5 | Octreotide, Pasireotide, Vapreotide | Metastatic carcinoid tumors, Cushing’s disease, esophageal variceal bleeding |
| 17:45360730-C (*) | 0.0556 | 0.1316 | 0.1538 | 0.1214 | 0.1059 | 0.0928 | ITGB3 | Abciximab, Antithymocyte globulin, Eptifibatide, Tirofiban | Cardiac ischemic complications, myocardial infarction, acute coronary syndrome |
| 20:43255220-C | 0.142 | 0.0842 | 0.0897 | 0.0643 | 0.0882 | 0.0825 | ADA | Adenosine, Dipyridamole, Edetic Acid, Nelarabine, Pentostatin, Theophylline, Vidarabine | Tachycardia, coronary artery disease, stroke, leukemia, etc. |

**Table S11.** List of variants in drug binding domains found to have a fold increase in allele frequency equal or higher than 1.5 comparing Roma and non-Roma. Genomic position, minor allele, MAF for each population, gene, drug reported to bind to the affected domain (Dopazo et al. 2016), and drug indication (according to DrugBank and PubChem databases) are shown. (*) variants with known Association drug phenotype annotated in PharmGKB.

| **Variant** | **Allele** | **SA** | **EUR** | **UN** |
| --- | --- | --- | --- | --- |
| **20:43255220** | Minor | 3 | 17 | - |
|  | Major | 32 | 69 | 3 |
| **9:35679251** | Minor | - | 8 | - |
|  | Major | 53 | 61 | 2 |
| **13:103718308** | Minor | 1 | 6 | - |
|  | Major | 50 | 63 | 4 |
| **11:92715117** | Minor | 2 | 2 | - |
|  | Major | 40 | 77 | 3 |
| **14:95054012** | Minor | 7 | 5 | - |
|  | Major | 26 | 82 | 4 |
| **14:95053863** | Minor | 7 | 5 | - |
|  | Major | 26 | 82 | 4 |
| **1:75175886** | Minor | 19 | 8 | 1 |
|  | Major | 14 | 78 | 4 |
| **3:8809222** | Minor | 2 | 24 | 1 |
|  | Major | 29 | 64 | 4 |
| **15:75012987** | Minor | - | 7 | - |
|  | Major | 18 | 93 | 6 |
| **1:20246876** | Minor | 1 | 5 | - |
|  | Major | 45 | 73 | - |
| **17:45360730** | Minor | 1 | 5 | - |
|  | Major | 54 | 64 | - |
| **10:101595996** | Minor | - | 4 | - |
|  | Major | 45 | 75 | - |
| **6:39034072** | Minor | 3 | 10 | - |
|  | Major | 49 | 60 | 2 |
| **1:53679229** | Minor | - | 12 | 1 |
|  | Major | 31 | 80 | - |
| **11:20648364** | Minor | - | 7 | 1 |
|  | Major | 29 | 87 | - |
| **8:143961102** | Minor | 4 | 4 | - |
|  | Major | 29 | 87 | - |
| **15:75012985** | Minor | 2 | 2 | - |
|  | Major | 21 | 98 | 1 |
| **Variant** | **Genotype** | **SA-SA** | **EUR-EUR** | **EUR-SA** |
| **6:102516260** | Minor hom | - | - | - |
|  | Het | - | 3 | 1 |
| **10:129868686** | Minor hom | - | - | - |
|  | Het | 1 | - | 3 |
| **7:139715645** | Minor hom | - | - | - |
|  | Het | - | - | 1 |
| **13:78475230** | Minor hom | - | 1 | - |
|  | Het | - | 2 | 1 |
| **16:1129193** | Minor hom | - | 1 | - |
|  | Het | - | 5 | 3 |

**Table S13.** Local ancestry assignment (SA: South Asian, EUR: European, UN: Unassigned) for the minor and major alleles of the variants described in Table S11. When the variant was filtered out with MAF<1% filter, only haplotype ancestry information could be retrieved (Minor hom: Minor homozygote; Het: Heterozygote). Data from the merged WES-array dataset.

| **Variant** | **Allele** | **SA** | **EUR** | **UN** |
| --- | --- | --- | --- | --- |
| **rs717620** | Minor | 6 | 18 | - |
|  | Major | 33 | 59 | 8 |
| **rs4149056** | Minor | 1 | 16 | 1 |
|  | Major | 31 | 73 | 2 |
| **rs10509681** | Minor | 2 | 7 | - |
|  | Major | 31 | 84 | - |
| **rs8192709** | Minor | - | 5 | - |
|  | Major | 35 | 82 | 2 |
| **rs1800460** | Minor | - | 3 | - |
|  | Major | 45 | 72 | 4 |
| **rs1799814** | Minor | - | 7 | - |
|  | Major | 18 | 93 | 6 |
| **rs316019** | Minor | 9 | 16 | 1 |
|  | Major | 30 | 68 | - |
| **rs1058930** | Minor | - | 15 | - |
|  | Major | 33 | 76 | - |
| **rs1048943** | Minor | 2 | 2 | - |
|  | Major | 21 | 99 | - |
| **rs2282143** | Minor | 3 | - | - |
|  | Major | 35 | 86 | - |
| **rs4244285** | Minor | 13 | 8 | - |
|  | Major | 19 | 84 | - |
| **rs12208357** | Minor | - | 3 | - |
|  | Major | 38 | 83 | - |
| **Variant** | **Genotype** | **SA-SA** | **EUR-EUR** | **EUR-SA** |
| **rs34059508** | Minor hom | - | - | - |
|  | Het | - | 2 | - |

**Table S15.** Local ancestry assignment (SA: South Asian, EUR: European, UN: Unassigned) for the minor and major alleles of the variants increased in Roma described in Table 3. When the variant was filtered out with MAF<1% filter, only haplotype ancestry information could be retrieved (Minor hom: Minor homozygote; Het: Heterozygote). Data from the merged WES-array dataset.

**References**

1000 Genomes Project Consortium. 2012. An integrated map of genetic variation from 1,092 human genomes. Nature. 491(7422):56.

Ali-Khan SE, Krakowski T, Tahir R, Abdallah •, Daar S. 2011. The use of race, ethnicity and ancestry in human genetic research. Hugo J.

Caulfield T, Fullerton SM, Ali-Khan SE, Arbour L, Burchard EG, Cooper RS, Hardy BJ, Harry S, Hyde-Lay R, Kahn J, et al. 2009. Race and ancestry in biomedical research: Exploring the challenges. Genome Med. 1(1).

Cornell S, Hartmann D. 2007. Ethnicity and Race: Making Identities in a Changing World. Pine Forge Press.

Dopazo J, Amadoz A, Bleda M, Garcia-Alonso L, Alemán A, García-García F, Rodriguez JA, Daub JT, Muntané G, Rueda A, et al. 2016. 267 Spanish Exomes Reveal Population-Specific Differences in Disease-Related Genetic Variation. Mol Biol Evol. 33(5):1205–1218.

Liao Y, Wang J, Jaehnig EJ, Shi Z, Zhang B. 2019. WebGestalt 2019: gene set analysis toolkit with revamped UIs and APIs. Nucleic Acids Res. 47(W1):W199–W205.

Mathieson I, Scally A. 2020. What is ancestry? PLoS Genet. 16(3):6–11.

Mersha TB, Abebe T. 2015. Self-reported race/ethnicity in the age of genomic research: its potential impact on understanding health disparities. Hum Genomics. 9.

Milanov I, Kmetski T, Lyons KE, Koller WC. 2000. Prevalence of Parkinson’s disease in Bulgarian Gypsies. Neuroepidemiology. 19(4):206–209.

National Cancer Institute. NCI’s Dictionary of Genetic Terms. Accessed online 2021: https://www.cancer.gov/publications/dictionaries/genetics-dictionary.

Peterson RE, Kuchenbaecker K, Walters RK, Chen CY, Popejoy AB, Periyasamy S, Lam M, Iyegbe C, Strawbridge RJ, Brick L, et al. 2019. Genome-wide Association Studies in Ancestrally Diverse Populations: Opportunities, Methods, Pitfalls, and Recommendations. Cell. 179(3):589–603.

Race Ethnicity and Genetics Working Group. 2005. The use of racial, ethnic, and ancestral categories in human genetics research. Am J Hum Genet. 77(4):519–532.

Richards CS, Bale S, Bellissimo DB, Das S, Grody WW, Hegde MR, Lyon E, Ward BE. 2008. ACMG recommendations for standards for interpretation and reporting of sequence variations: Revisions 2007. Genet Med. 10(4):294–300.

Richards S, Aziz N, Bale S, Bick D, Das S, Gastier-Foster J, Grody WW, Hegde M, Lyon E, Spector E, et al. 2015. Standards and guidelines for the interpretation of sequence variants: A joint consensus recommendation of the American College of Medical Genetics and Genomics and the Association for Molecular Pathology. Genet Med. 17(5):405–424.

Shaw-Smith C. 2006. Oesophageal atresia, tracheo-oesophageal fistula, and the VACTERL association: Review of genetics and epidemiology. J Med Genet. 43(7):545–554.

Stanley CM, Sunyaev SR, Greenblatt MS, Oetting WS. 2014. Clinically Relevant Variants - Identifying, Collecting, Interpreting, and Disseminating: The 2013 Annual Scientific Meeting of the Human Genome Variation Society. Hum Mutat. 35(4):505–510.
